# Combined effects of sleep quality and depression on quality of life in patients with type 2 diabetes

**DOI:** 10.1186/s12875-016-0435-x

**Published:** 2016-04-05

**Authors:** Pan Zhang, Peian Lou, Guiqiu Chang, Peipei Chen, Lei Zhang, Ting Li, Cheng Qiao

**Affiliations:** School of Public Health, Xuzhou Medical University, Xuzhou, China; Department of Control and Prevention of Chronic Non-communicable Diseases, Xuzhou Center for Disease Control and Prevention, School of Public Health, Xuzhou Medical University, 42 West Erhuan Road, Xuzhou, Jiangsu China; Department of Control and Prevention of Chronic Non-communicable Diseases, Xuzhou Center for Disease Control and Prevention, Xuzhou, China

**Keywords:** Type 2 diabetes mellitus, Sleep quality, Depression, Diabetes specificity quality of life scale, Interaction, Relative excess risk of interaction, Attributable proportion, Synergy index

## Abstract

**Background:**

Poor sleep quality and depression negatively impact the health-related quality of life of patients with type 2 diabetes, but the combined effect of the two factors is unknown. This study aimed to assess the interactive effects of poor sleep quality and depression on the quality of life in patients with type 2 diabetes.

**Methods:**

Patients with type 2 diabetes (*n* = 944) completed the Diabetes Specificity Quality of Life scale (DSQL) and questionnaires on sleep quality and depression. The products of poor sleep quality and depression were added to the logistic regression model to evaluate their multiplicative interactions, which were expressed as the relative excess risk of interaction (RERI), the attributable proportion (AP) of interaction, and the synergy index (S).

**Results:**

Poor sleep quality and depressive symptoms both increased DSQL scores. The co-presence of poor sleep quality and depressive symptoms significantly reduced DSQL scores by a factor of 3.96 on biological interaction measures. The relative excess risk of interaction was 1.08. The combined effect of poor sleep quality and depressive symptoms was observed only in women.

**Conclusions:**

Patients with both depressive symptoms and poor sleep quality are at an increased risk of reduction in diabetes-related quality of life, and this risk is particularly high for women due to the interaction effect. Clinicians should screen for and treat sleep difficulties and depressive symptoms in patients with type 2 diabetes.

## Background

With an alarmingly increasing rate worldwide, diabetes mellitus in adults is predicted to reach approximately 592 million by 2035 [1]. In China, the prevalence of diabetes is also increasing rapidly, affecting approximately 11.6 % of the Chinese population and making the disease an important public health problem [2]. Patients with diabetes require lifelong and comprehensive self-management, including dietary restrictions, frequent physical exercise to maintain optimal weight, medication compliance, and blood-glucose monitoring [3]. In such a long-term self-management process, many patients experience emotional burdens and poor sleep quality in response to these prolonged requirements. Burdens include worry about complications, fear of hypoglycemia, feelings of guilt regarding uncontrolled blood glucose, and depressed mood [4].

Depression is one of the most common co-morbidities among patients with diabetes [5, 6], and those who have diabetes with comorbid depression tend to have more diabetes-related complications [7], which also lead to greater dysfunction [8] and worse quality of life [9–12] compared with patients who have normal mood states. Depression with diabetes also represents increased health care costs [5, 6], and even prompts a higher rate of premature death when compared with either condition alone [13]. Poor sleep quality is another common feature of type 2 diabetes [14]. It has been reported that more than half of patients with diabetes have poor sleep quality [15]. Poor sleep quality was significant in predicting diabetes-related quality of life after controlling for age, years since diabetes diagnosis, number of comorbidities, number of diabetic complications, insulin use, and depressive symptoms [15, 16], and worse physical, mental, and functional outcomes in adults with type 2 diabetes [17].

Although poor sleep quality and depression have been shown to be individually correlated to quality of life in people with type 2 diabetes, to the best of our knowledge, no studies have been done on the interaction of sleep quality and depressive symptoms as they relate to quality of life in patients with type 2 diabetes. Hence, the aims of the current study were twofold. A first aim was to examine the joint effects of poor sleep quality and depressive symptoms on quality of life in adult patients with type 2 diabetes in primary care in China. The prevalence and severity of poor sleep quality and depression symptoms may differ according to sex [6, 18]. Therefore, a second aim of this study was to assess the interaction of sleep quality combined depression symptom on quality of life in patients with type 2 diabetes according to gender.

## Methods

### Design and sample

The method of patient selection has been described previously [19]. Briefly, from August to December 2012, 944 type 2 diabetes patients from 37 communities met the criteria and were enrolled in an observational study. Diagnostic criteria of patients with diabetes was consistent with the recommendations of the American Diabetes Association [20]. The following exclusion criteria were used by our study: 1) diagnosis of type 2 diabetes was made by a physician at least six months prior to study enrollment; 2) type 1 diabetes; 3) painful diabetic sensory neuropathology; 4) diagnosed sleep disorders prior to diabetes; 5) mental illness or use of any psychotropic medication; 6) working night shifts in the past 3 months or travelling across time zones within the past month; 7) other endocrine disorders, such as, thyroid disease or chronic use of glucocorticoids; 8) age <18 years; 9) pregnancy or lactation.

Written informed consent was obtained from all participants. The study was approved by the ethics committee of Xuzhou Center for Disease Control and Prevention (2011710). The procedures followed were in accordance with the standards of the ethics committee of Xuzhou Center for Disease Control and Prevention and with the Declaration of Helsinki (1975, revised 2000).

### Sleep quality

Sleep quality was measured using the Pittsburgh Sleep Quality Index (PSQI) [21]. The PSQI is a 19-item self-report measure of sleep quality and degree of sleep difficulties over the past month and contains seven component scales: sleep quality, sleep latency, sleep duration, sleep efficiency, sleep disturbances, use of sleep medication, and daytime dysfunction. The 7 component scores are then summed to yield a global PSQI score with a range of 0–21; higher scores indicate worse sleep quality. The Chinese version of the PSQI used in this study has been approved by the original PSQI authors. A global PSQI score >7 had a diagnostic sensitivity of 98.3 % and specificity of 90.2 % in distinguishing normal subjects from patients with sleep quality problems. The good clinical practice and psychometric properties of the PSQI suggest its usefulness in both Chinese psychiatric clinical practice and research activities [22]. In our study, a PSQI score <7 and ≥ 7 were defined as ‘good sleep quality’ and ‘poor sleep quality’, respectively.

### Self-rating Depression Scale

The Self-rating Anxiety Scale (SAS) and Self-rating Depression Scale (SDS) assessed anxiety and depression [23, 24]. Both scales have demonstrated adequate reliability and validity for the Chinese population [25]. Each of the two scales included 20 questions, and each question was scored on a 4-point frequency scale (1, rarely; 2, some of the time; 3, very often/often; 4, almost always). The total score from the 20 questions was multiplied by 1.25, with the integer score as a standard score. Cutoffs for both anxiety and depression were standard scores ≥50.

### Other variables

Age, gender, marital status, physical activities, net household income, level of education, cigarette smoking, alcohol consumption, years since diabetes diagnosis, number of comorbidities, number of diabetic complications, and insulin use were assessed using a self made questionnaire with face to face interview at their homes by our study group. The number of diabetic complications was determined by assessing whether participants reported their diagnosed coronary artery disease, peripheral vascular disease, stroke, nephropathy, retinopathy, or neuropathy. All fasting venous blood samples were drawn between 8:00 AM and 9:00 AM. HbA1c levels were assayed using high-performance liquid chromatography (BIO-RAD Diagnostic Group, CA, USA) by our study group. We used the level of HbA1c as the index for glycemic control. HbA1c ≤6.5 % (48 mmol/mol) was defined as good glycemic control, based on the Chinese Type 2 Diabetes Prevention and Control 2010 Guidelines, while a level >6.5 % was considered poor glycemic control [26]. Body height (to the nearest 0.1 cm) and weight (to the nearest 0.1 kg) in light indoor clothing were measured by our group members at clinics. Body mass index (BMI; in kg/m^2^) was calculated, and categorized as underweight (<18.5 kg/m^2^), normal weight (18.5−23.9 kg/m^2^), or overweight/obese (≥24.0 kg/m^2^) [27].

### Diabetes Specificity Quality of Life Scale (DSQL)

Quality of life was measured using the Diabetes Specificity Quality of Life Scale (DSQL), which is a validated questionnaire developed by Chen et al. [28]. to assess life quality of type 2 diabetes patients in China. The scale includes 24 items with 4 domain scores that reflect problems with quality of life in the areas of Physiology, Psychology, Social, and Therapy. The sum of the scores for these 4 components produces a global quality of life score with a range of 24–120 points, where higher scores indicate worse quality of life. A DSQL score <40 had a diagnostic sensitivity of 94.5 % and specificity of 91.0 % in differentiating good from poor quality of life [28].

### Statistical analysis

The computer-based analysis program SPSS version 13.0 was used for all statistical analyses. Differences in continuous variables were tested using the F-test or the Student *t*-test, and differences in categorical variables were assessed using the Pearson *χ*^2^ test. The DSQL score, which represented quality of life was used as the dependent variable. The associations of sleep quality and depressive symptoms with patients’ quality of life were determined using binary logistic regression. The results were stratified by sleep quality (good versus poor) and depression (having depressive symptoms versus not), and were adjusted for age (continuous), sex, educational level (less than high school, high school, or higher), marital status, physical activities (yes or no), net household income (continuous), cigarette smoking (yes or no), alcohol consumption (yes or no), years since diabetes diagnosed (continuous), comorbidities (yes or no), diabetic complications (yes or no), anxiety (yes or no), insulin use (yes or no), and glycemic control (yes or no). A cross-product interaction term was included in the logistic regression model to assess multiplicative interactions. Odds ratios (ORs) and 95 % confidence intervals (CIs) were calculated using the contrast statement in SPSS 16.0. Variance was calculated using the Taylor series linearization method, which leads to an asymptotically unbiased estimate. All associations reported were statistically significant (*P* <0.05) using two-tailed tests.

To estimate the biological interaction between sleep quality and presence or absence of depression, we created three new variables: 1) PSQI global score <7 = no and depression = yes; 2) PSQI global score ≥7 = yes and depression = no; and 3) PSQI global score ≥7 = yes and depression = yes [29, 30].

The relative excess risk due to interaction (RERI), the attributable proportion due to interaction (AP), and the synergy index (S) were used to estimate biological interactions [30]. The RERI is the excess risk attributed to interaction relative to the risk without exposure. AP refers to the attributable proportion of disease caused by interaction in subjects with both exposures. S is the excess risk from both exposures when there is a biological interaction relative to the risk from both exposures without interaction. In the absence of additive interactions, RERI and AP are equal to 0 [31]. The current study refined the criteria as either a statistically significant RERI >0, AP >0, or S >1 to indicate biological interactions.

Subgroup analysis: A final set of logistic regression analyses were performed to assess whether the association between sleep quality and depression symptom level would vary as a function of gender. Regression analyses and interaction analyses were conducted for female and male subjects separately.

## Results

### General characteristics of participants

The mean age of participants was 64.1 ± 10.2 years, and 61.3 % were female. 56 patients were unanswered, there was accounted for 5.6 %. The mean age of non-respondents was 63.8 ± 10.1 years, and 59.9 % were female. There were no significant differences between participants and non-respondents (*P* >0.05). The average DSQL was 50.7 ± 12.9, and males had lower DSQL scores than did females (48.8 ± 12.7 vs 51.9 ± 13.1, *P* <0.01). A total of 19.9 % of the sample reported having good quality of life. Men were more likely to report having good quality of life than were women (32.3 % vs. 12.1 %, *P*<0.001). Within the sample, 17.3 % were smokers, 12.0 % were alcohol drinkers, 64.2 % did not have any comorbidity, and 12.4 % were treated with insulin. Participants had an average duration of 5.6 ± 5.1 years since type 2 diabetes had been diagnosed. The distribution of general characteristics of participants was presented in Table 1.

### Rates of poor sleep quality and depressive symptoms

The prevalence of poor sleep quality of the participants was 33.6 %. Women were slightly more likely than men to report having poor sleep quality (46.1 % vs. 37.8 %). A large fraction of participants, 40.1 %, had depressive symptoms, and 65.5 % had anxiety symptoms. Women had more depressive symptoms (43.4 % vs. 35.1 %) and anxious symptoms (72.4 % vs. 54.5 %) than did men. The rate of poor sleep quality was 21.3 % (40/188) in patients with good quality of life and 48.3 % (365/756) in patients with poor quality of life (*χ*^2^ = 44.82, *P* <0.01). The prevalence of depressive symptoms was 26.6 % (50/188) in patients with good quality of life and 43.5 % (329/756) in patients with poor quality of life (*χ*^2^ = 17.94, *P* <0.01). The rates of poor sleep quality and depressive symptoms were lower in patients with good quality of life than in patients with poor quality of life, regardless of gender (Table 1).

**Table 1:** The distribution of general characteristics of quality of life by gender

Variables	Men (*n* = 365)			Women (*n* = 579)		
	Good	Poor	*P*	Good	Poor	*P*
	*n* = 118	*N* = 247		*n* = 70	*n* = 509	
Age >60	62.1 ± 8.7	65.6 ± 10.6	0.002	61.6 ± 9.8	65.5 ± 11.1	0.006
Above high school	14(11.86)	39(15.79)	0.319	5(7.14)	27(5.30)	0.725
No spouse	18(15.25)	56(22.67)	0.099	8(11.43)	73(14.34)	0.51
Regular exercise	105(88.98)	193(78.14)	0.012	60(85.71)	396(68.39)	0.129
Income below population average (%)	49(41.53)	125(50.60)	0.104	46(65.71)	269(52.85)	0.043
BMI	24.1 ± 2.5	23.8 ± 2.9	0.335	24.0 ± 2.5	23.8 ± 2.8	0.571
Median of disease duration (years)	3.8 ± 3.6	6.2 ± 5.1	<0.01	3.8 ± 3.7	6.1 ± 5.4	<0.01
Comorbidities	23(19.49)	107(43.32)	<0.01	24(34.29)	184(36.15)	0.761
Complications	10(8.47)	71(28.74)	<0.01	3(4.29)	155(30.45)	<0.01
Smokers	30(25.42)	105(42.51)	0.002	7(10.00)	21(4.13)	0.064
Drinkers	26(22.03)	61(24.70)	0.577	8(11.43)	18(3.54)	0.007
Depression	33(27.97)	95(38.46)	0.049	17(24.29)	234(45.97)	0.001
Anxiety	37(31.36)	162(65.59)	<0.01	49(70.00)	370(72.69)	0.637
Using insulin	4(3.39)	37(14.98)	0.001	2(2.86)	74(14.54)	0.007
HbA1c <6.5 %	36(30.51)	35(14.17)	<0.01	24(34.29)	69(13.56)	<0.01
PSQI ≥ 8	25(21.19)	113(45.75)	<0.01	15(21.43)	252(49.51)	<0.01

*BMI* Body mass index; *HbA1c* Glycated hemoglobin; *DSQL* Diabetes Specificity Quality of Life Scale. Comorbidity refers to disease accompanying diabetes; Complication refers to a disease caused by diabetes; *PSQI* Pittsburgh Sleep Quality Index

### Correlations of PSQI scores and SDS scores with DSQL

The correlations between PSQI and DSQL scores, and between SDS and DSQL scores were positive, with correlation coefficients of 0.386, 0.364 (all *P* <0.001), respectively. Separate correlation analyses were conducted for male and female subjects. For men, the correlation coefficients between PSQI scores and DSQL scores, and between SDS scores and DSQL scores, respectively, were 0.395, 0.337 (all *P* <0.001). For women, these values were 0.341, 0.371 (all *P* <0.001), respectively.

### Biological interaction of sleep quality and depressive symptoms in quality of life of patients with type 2 diabetes

The results from the multiple logistic regression models are presented in Table 2 to reveal interaction using a combined effects method. Diabetic patients with poor sleep quality and no depressive symptoms had a 2.77-fold higher risk of reduced health status as indicated by the DSQL score compared with patients who had neither poor sleep quality nor depressive symptoms. Only depressive patients who were good sleepers also had reduced health status compared with that of patients who had neither poor sleep nor depressive symptoms (OR: 1.15; 95 % CI: 1.05–1.65). Diabetic patients who had both poor sleep quality and depressive symptoms had a 3.96-fold increased risk of reduced health status as measured by the DSQL, compared with those who neither slept poorly nor had depression. Separate regression analyses were conducted for male and female participants. For men, poor sleep quality played a greater role in reducing DSQL than did depressive symptoms; for women, depressive symptoms played the larger role. The combined effect of poor sleep quality and depressive symptoms produced greater reductions in DSQL for women than for men (see Table 2).

### Sensitivity analysis

There was an additive interaction between poor sleep quality and presence of depressive symptoms (RERI, 1.08; 95 % CI: 0.74–2.01) in the sample at large (Table 3): the OR of reduced DSQL score in patients with both poor sleep quality and depressive symptoms was 1.08 times higher than that of good sleepers who did not have depression, with 27 % of declining health status attributed to the interaction of sleep quality and depression. For men, there was no interaction between poor sleep quality and depressive symptoms (RERI, −0.42; 95 % CI: −1.12–0.01). For women, there was an additive effect (RERI, 1.85; 95 % CI: 1.23–3.01), with 44 % of declining health status attributed to the interaction between poor sleep quality and depression.

**Table 2:** Odds ratios for the association between sleep quality and quality of life by depressive symptom among patients with type 2 diabetes

Sleep quality	Depressive symptoms	Quality of Life								
		Men			Women			Total		
		Good (*n* = 118)	Poor (*n* = 247)	OR(95%*CI*)	Good (*n* = 70)	Poor (*n* = 509)	OR(95%*CI*)	Good (*n* = 188)	Poor (756)	OR(95%*CI*)
Good	<50	76	107	1	47	212	1	123	319	1
Good	≥50	17	27	1.10(1.03 ~ 1.55)	8	45	1.19(1.07 ~ 1.62)	25	72	1.15(1.05 ~ 1.65)
Poor	<50	9	45	3.27(2.42 ~ 4.78)	6	63	2.11(1.83 ~ 5.16)	15	108	2.77(1.42 ~ 6.28)
Poor	≥50	16	68	2.95(2.58 ~ 5.14)	9	189	4.15(3.12 ~ 9.96)	25	257	3.96(1.58 ~ 5.74)

^§^Models were adjusted for age, sex, disease duration, marital status, income, education level, smoking status, comorbidity, anxiety

**Table 3:** Measures for estimating biological effects on the combination of sleep quality and depressive symptoms on the reduction of quality of life in patients with type 2 diabetes

Measures of biological interaction	Estimate (95 % CI)		
Sleep quality versus Depression			
	Men	Women	Total
RERI	−0.42(−1.12-0.01)	1.85(1.23-3.01)	1.08(0.74-2.01)
AP	−0.14(−0.31-0.19)	0.44(0.25-0.67)	0.27(0.13-0.51)
S	0.82(0.27-1.56)	2.42(1.48-4.11)	1.57(1.12-2.17)

Reference group is sleep quality score <8 and depressive symptom score < 50

Models were adjusted for age, sex, education level, marital status, exercise, income, BMI, disease duration, co-morbidity, complication, smoking. drinking, insulin use, and anxiety

## Discussion

This study produced three main findings. First, an interaction effect of poor sleep quality and depressive symptoms in increasing DSQL scores of type 2 diabetes patients was observed. Second, this interaction only existed for women. Third, poor sleep quality alone produced a greater addition of DSQL scores in our sample than did depressive symptoms alone.

Sleep disorders and depression are in close relationship. An epidemiological survey has shown that more than 90 % of patients with depression will have a sleep disorder, and that patients with depression usually have difficulties falling asleep, awaken frequently during the night, awaken early, and experience non-refreshing sleep [32, 33]. Conversely, sleep disorders can also cause depression: in one study, 41 % of patients experienced a sleep disorder before a major depressive disorder, and approximately 29 % of patients experienced sleep disorders and depression simultaneously [32, 34]. In the present study, we found for the first time a combined effect of poor sleep quality and depression in DSQL score additions of patients with type 2 diabetes; i.e., DSQL scores were higher for patients with both poor sleep quality and depressive symptoms than for patients experiencing only one of these difficulties.

Depression and poor sleep quality may affect quality of life of patients with diabetes through either pathophysiologic or behavioral pathways. Pathophysiologic pathways of both poor sleep and depression involve dysregulation in the fronto-limbic system and reductions in hippocampal volume [33, 35]. These in turn lead to dysregulation of the hypothalamic-pituitary adrenal axis and the autonomic nervous system, inducing increases in glucocorticoids secretion, alterations in glucose transport function, and increased immunoinflammatory activation [36], which results in elevated blood glucose. Behavioral pathways of depression and poor sleep include insufficient diabetes self care, poor diet, and persistent poor glycemic control [37–40]. Taken together, these factors produce a vicious cycle that decreases the quality of life of patients with type 2 diabetes.

When the interaction between poor sleep quality and depression symptoms was assessed separately for men and women, an important gender difference emerged: this interaction only produced lower quality of life in women. The hormone theory provides a useful framework for interpreting this finding. After the age of 30 years, estrogen decreases gradually in women [41] and results in a decline in well-being [42]. Decreased estrogen can also aggravate insomnia. According to this theory, lower quality of life results from a combination of factors including insomnia and the decline in well-being. An alternative explanation for this finding is the higher prevalence of poor sleep quality and depression in women than in men in our study.

Our study showed a strong negative association between depression and health-related quality of life in patients with type 2 diabetes, which consistent with previous reports [9–12]. Not surprisingly, we also found that PSQI and DSQL scores were strongly associated. Poor sleep quality of patients with type 2 diabetes has been shown to predict a lower quality of life [15–17]. However, poor sleep quality produced greater reductions in DSQL scale than did depressive symptoms. This may support that sleep quality has an important role in mediating the way in which psychological distress affects quality of life for those with diabetes [43].

Strengths of our study include the homogeneity of the random sample, large sample size, and the community-based nature of the sample. Limitations include the reliance on self-reported measures of poor sleep quality and depression levels. Also, owing to the cross-sectional nature of this sample, the temporal order of causality cannot be argued conclusively. Third, although we used reliable and valid measures of anxiety and depression, these measures did not constitute a clinical diagnosis of poor sleep quality or of depression disorder. Fourth, we were unable to adjust for other confounds, such as hypoglycemia. Fifth, these findings were only derived from Chinese patients and need to be replicated in other ethnic populations.

### Implications

Despite these limitations, we believe that our results have great significance for public health. In fully adjusted models, we estimated that 27 % (44 % in women) of the addition in DSQL of patients with type 2 diabetes can be explained by an interaction between poor sleep quality and depressive symptoms. The finding suggests that clinicians should screen for and treat depression complaints and poor sleep quality in these patients. Efficacious behavioral and pharmacologic treatments exist for both depression disorders and poor sleep quality [44, 45]. Identification and implementation into routine health care for integrated health services for comorbid depression and diabetes in different types of service and in different countries [46]. The application of either a depression- or sleep-focused intervention could produce relief in both symptoms, thereby maximizing improvements in patients’ quality of life. Given the difficulty in treating poor sleep quality or depression in certain patients, recommendations that help prevent poor sleep quality and depression are an inexpensive and practical means of improving DSQL for patients with type 2 diabetes.

## Conclusions

Our study showed a strong negative association between depression and health related quality of life in patients with type 2 diabetes. And we found that PSQI and DSQL scores were strongly associated. Patients with both depressive symptoms and poor sleep quality are at an increased risk of reduction in diabetes-related quality of life. Clinicians should screen for and treat sleep difficulties and depressive symptoms in patients with type 2 diabetes.

## Abbreviations

AP: the attributable proportion of interaction
BMI: body mass index
DSQL: the Diabetes Specificity Quality of Life scale
HbA1c: glycated haemoglobin
PSQI: the Pittsburgh Sleep Quality Index
RERI: the relative excess risk of interaction
S: the synergy index
SAS: The Self-rating Anxiety Scale
SDS: The Self-rating Depression Scale
